# Bee Products for Poultry and Rabbits: Current Challenges and Perspectives

**DOI:** 10.3390/ani13223517

**Published:** 2023-11-14

**Authors:** Ayman Abd El-Aziz, Mahmoud Abo Ghanima, Daniel Mota-Rojas, Anjum Sherasiya, Francesca Ciani, Karim El-Sabrout

**Affiliations:** 1 Department of Animal Husbandry and Animal Wealth Development, Faculty of Veterinary Medicine, Damanhour University, Damanhour 22511, Egypt; ayman.sadaka@vetmed.dmu.edu.eg (A.A.E.-A.);; 2 Neurophysiology of Pain, Behavior and Assessment of Welfare in Domestic Animals, DPAA, Xochimilco Campus, Universidad Autónoma Metropolitana (UAM), Mexico City 04960, Mexico; 3 Veterinary World, Wankaner 363621, Gujarat, India; 4 Department of Veterinary Medicine and Animal Production, University of Naples Federico II, 80138 Naples, Italy; ciani@unina.it; 5 Poultry Production Department, Faculty of Agriculture, Alexandria University, Alexandria 21545, Egypt

**Keywords:** antioxidant, bee venom, chicken, heat stress, immunity, rabbit, reproduction, sustainability, welfare

## Abstract

**Simple Summary:**

Small-livestock products, such as poultry and rabbit meats, are essential to global animal food production. Poultry and rabbit production has recently experienced many challenges, including climate change, disease spread, and antibiotic misuse. Bee products have emerged as promising natural alternatives to antibiotics due to their nutritional and therapeutic properties. This review aims to present an overview of the possible advantages of using different bee products, separately or combined, in poultry and rabbit farms. The results indicate that bee products can positively affect poultry and rabbit’s physiological traits, production, reproduction, health/immunity, and product quality. Therefore, bee products have great potential as a safe and effective addition to poultry and rabbit production.

**Abstract:**

Poultry and rabbit production are important and rapidly growing agricultural subsectors, particularly in several developing countries. To ensure the sustainability of poultry and rabbit production, realistic poultry and rabbit farming practices must be improved. Apitherapy is a traditional alternative medicine that involves the prevention and treatment of some diseases with several bee products including propolis, royal jelly, pollen, and venom. More feeding investigations on the numerous benefits of bee products for poultry and rabbits are crucial to be addressed. Poultry and rabbit production has recently experienced numerous challenges, including climate change, disease spread, and antibiotic misuse. Improving animal welfare, health, and production is a top priority for all livestock farms, as is supplying consumers with safe and healthy products. Therefore, this review aims to collect and investigate recent relevant literature on the use of bee products, as feed additives, drinking water supplements, and injections, for poultry and rabbits to improve animal health and production. From the current findings, bee products can improve the growth and immunological performance of small-livestock animals, such as poultry and rabbits, by activating digestive enzymes, maintaining microbial balance, and promoting vitamin synthesis. Therefore, bee products could be a promising natural alternative to growth promoters, reproductive stimulants, and immunological enhancers in poultry and rabbit farms to provide safe and healthy products for humans.

## 1. Introduction

Small-livestock farming, such as poultry and rabbits, is an important component of food production, helping to meet the expanding global need for protein-rich foods. Consumers have expressed considerable interest in enhancing animal welfare and product quality. In several developed countries, they have also become interested in functional, healthy, and organic foods [1]. However, poultry and rabbit industries have recently experienced numerous challenges, including climate change, disease spread, and antibiotic misuse. Antibiotics have been widely used in poultry and rabbit farms for decades to increase animal productive traits [2,3,4,5]. Overuse of antibiotics as growth promoters in poultry and rabbit farms has resulted in several adverse effects, including the development of microbial resistance and transference [5,6,7], and the residues remaining in the consumed product. Various strategies have been applied to deal with this misuse of synthetic drugs; one of these strategies is to use natural substances such as honeybee products in poultry and rabbit farms to improve animal welfare and produce healthier products. 

Apitherapy is a subspecialty of complementary medicine that involves using *Apis mellifera* L. produced materials, including honey and venom, for the treatment of various diseases. Bee products not only provide essential nutrients, but also have medicinal properties [3,4] (Table 1). Thus, they can be applied in animal diets, drinking water, and by muscular injection to improve productivity, immunity, and health [3,4,8,9]. Bee products can also be administered for better chick quality through the *in ovo* technique, as a recent aspect of poultry farming [10,11]. Injecting 10 µg of bee venom extract (melittin)/egg on day 18 of incubation increased the post-hatch chick’s weight and enhanced immunological indicators (immunoglobulins, T cells, and B cells) [12]. Furthermore, bee products, particularly royal jelly and propolis, can promote some vitamin synthesis by providing the precursors required for this synthesis, enhancing their synthesis pathway, and improving organs generated [3,4,13]. However, bee products can be considered an excellent functional food to be added to poultry and rabbit diets, but to avoid harming animal’s digestive system or poisoning, it is always advisable to be cautious and introduce new foods gradually and moderately, as well as considering the animal’s age and safe doses because there are no available studies on the safe dosage limits of these substances for poultry and rabbits until now. In addition, some bee products contain anti-nutrients, which are chemical compounds that can interfere with nutrient absorption, such as bee honey that contains a tiny amount of cyanide [14] and bee venom that contains melittin, a peptide toxin [15].

**Figure 1:**
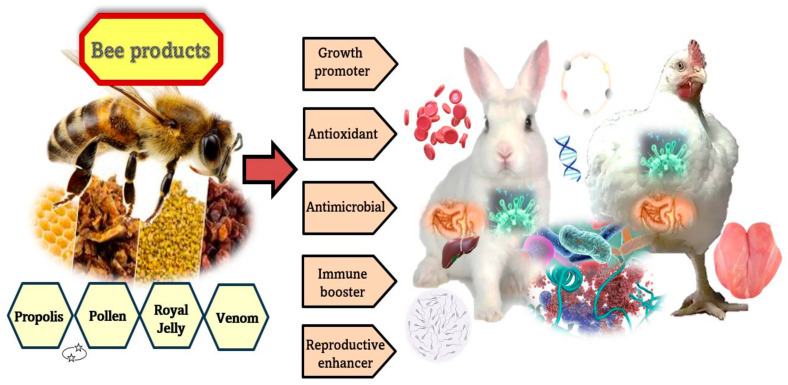
The potential role of bee products on poultry and rabbit performance.

The consumption of products that protect health appears as the primary preference of people. Every year, new studies that indicate the impact of bee products on animal products and global health are conducted, and the importance of these studies is gradually increasing. Many natural substances produced by honeybees, such as propolis, royal jelly, beeswax, venom, slum gum, and pollen have unique structures and high nutritional value with a variety of medicinal qualities [28]. Despite some of these substances being considered by-products, they are sources of bioactive compounds with relevant biological effects and significant impacts on animal physiological and productive performance [4,29,30]. Several studies have found that bee products, with a high content of essential amino acids, antioxidants, active enzymes, vitamins, minerals, antibacterial, antiprotozoal, and immunostimulant substances, can improve animals’ growth, meat quality, and immunological performance [3,4,31,32,33,34] (Figure 1). Rabie et al. [29] found that administering propolis (400 mg/kg diet) or bee-pollen (2 g/kg diet) to the broiler diets as well as adding bee-venom (2 mg/L water) to broiler drinking water is recommended instead of using antibiotics in broiler farms to increase body weight and improve blood biochemical indices. Bee products can also enhance animal fertility (males and females) by improving cryopreservation of gametes [34]. However, this study reviews and summarizes the recent progress of bee products’ nutritional and therapeutic effects on poultry and rabbit health, production, and welfare (Table 2), in addition to discussing several mechanisms underlying their primary constituents.

## 2. Potential Effects of Bee Products on Poultry and Rabbit Performance

### 2.1. Bee Propolis

Bee propolis (known as bee glue) is a natural bio-product rich in resins, phenolic acids, and flavonoids, besides containing active enzymes, minerals, and vitamins, which have significant antioxidant and antimicrobial activities [3,50,51,52]. Bee propolis has many positive effects on livestock animals, such as improving productive traits including feed efficiency, body weight, milk yield, and immunity [3,4,53].

Hassan et al. [54] reported that dietary inclusion of propolis in broiler diets (300 mg/kg diet) increased growth performance and immune response. This inclusion also improved some blood biochemical traits, such as total protein and globulin, while decreasing blood cholesterol and triglyceride concentrations. Serum biochemical profile, including total protein, globulin, total antioxidant capacity (TAC), aspartate aminotransferase (AST)/alanine aminotransferase (ALT), hormones (triiodothyronine (T_3_) and corticosterone), and cholesterol concentrations, is a good indicator of animal health and production because blood biochemical trait concentrations are related to several productive traits and immunological health performance [42,55,56,57]. Likewise, Dosoky et al. [35] revealed that propolis, at the level of 300 mg/kg diet, has positive effects on bird performance, antioxidative activity, and immunity. Additionally, Chegini et al. [58] indicated that the addition of 4 g/kg of propolis to broiler diets improved birds’ growth performance and nutrient digestibility under heat and overcrowding stresses. Propolis can also improve some carcass traits, such as dressing percentage, and meat quality, such as breast muscle color, if it is added with bee pollen [59]. It can also decrease total lipids, cholesterol, triglycerides, and malondialdehyde levels in the blood [35]. According to [60], propolis can be used as a natural and promising sanitizer for hatching chicken eggs since it reduces the microbial load on the eggshell and promotes embryo safety. Furthermore, Elsherif et al. [61] found that the mixture of castor extract (0.75 g/Kg) and propolis extract (0.75 g/Kg diet) could be the most effective immune booster for birds against Newcastle (ND) and Avian Flu (H5). It could also be an efficient and safe alternative to antibiotics in broiler farms. Moreover, Ivana et al. [55] observed that using propolis and bee pollen as supplements in broiler diets, either separately or in combination, results in more vital and healthier birds due to the high nutritional value of these substances, which positively affected the blood biochemical parameters of the birds. Propolis and bee pollen may benefit birds’ growth by increasing antioxidant capacity and enhancing immune-system defenses [62].

On the other hand, adding propolis to rabbit diets, at the level of 250–500 mg/kg of diet, lowered the colonization of *Salmonella* spp. and *Escherichia coli* in the cecum of treated rabbits [36]. Thus, this addition can improve growing rabbits’ immune response by positively impacting the microbiota in the cecum [36]. Similarly, Sierra-Galicia et al. [37] reported that propolis supplementation (50 μL/kg body weight) can work as a natural growth promoter in rabbits and prevent coccidiosis without affecting rabbit health and meat quality. 

El-Sherbiny [63] showed that the administration of propolis (10 mg/0.5 mL water) to rabbit bucks orally during summer months improved their reproductive performance and semen quality as determined by higher testosterone concentration, conception rate, libido, ejaculate volume, and sperm concentration, as well as enhanced liver functions. Furthermore, Attia et al. [64] found that propolis with bee pollen significantly increased does’ litter size (~39%) and milk yield (~43.5%), while decreased feed intake (~4.5%), as well as increased plasma total protein (~43%), albumin (~45.5%), and globulin (~40%). In the same manner, Sierra-Galicia et al. [65] reported that diets enriched/supplemented with propolis and bee pollen increased rabbits’ weight gain and total antioxidant capacity in blood serum while decreasing feed conversion rate. Aromatic acids, flavonoids, and phenolic compounds are the major substances that interact with the biological features of propolis [66,67]. Therefore, propolis and bee pollen, together, represent a powerful natural growth booster and immune enhancer for farm animals. Furthermore, adding an ethanolic extract of propolis to drinking water for rabbits suffering from chronic diarrhea resulted in decreased diarrhea duration and increased final body weight, particularly when combined with an herbal mixture like (*Rumex crispus*, *Potentilla anserina*, and *Polygonum aviculare*) [68].

### 2.2. Bee Pollen

Bee pollen grains (also called bee bread) are the cells of the male reproductive spore of flowers collected and combined with special enzymes and natural substances derived from bee salivary gland secretion [4,69]. It provides a variety of essential dietary components for both humans and animals. Due to the substantial quantity of proteins (~23%), essential amino acids (~10%), carbohydrates (~30%), vitamins, minerals, polyphenols, tannins, and essential fatty acids (~5%), bee pollen has been employed as a natural growth promoter and health booster for farm animals [70,71,72,73]. 

Bee pollen, according to [69], can increase an animal’s body weight by improving feed conversion and increasing the surface area of the duodenum, jejunum, and ileum’s intestinal villi [74]. Additionally, it may be the driving force behind better carcass and meat quality due to a decrease in fat deposition and an increase in amino acids. Likewise, Nemauluma et al. [75] reported that bee pollen inclusion in the starter diets (12 g/kg) has positive effects on growth performance and carcass yield without any adverse effect on the meat quality of broiler chickens. Abdelnour et al. [69] also reported that bee pollen supplementation (up to 20 g/kg diet) has positive effects on the productivity and health features of farm animals including its role as an antioxidant and production promoter. Bee pollen is rich in flavonoids and polyphenolic compounds, which have high antioxidant capacity due to their ability to scavenge free radicals via metal chelation [76]. According to Yıldız et al. [77], supplementation of bee pollen under stress lowered the oxidative stress indicators and increased the antioxidant system of animals. Moreover, Abuoghaba et al. [31] found that dietary supplementation of bee pollen (500 and 1000 mg/kg diet) to breeder hens improved several hatching and hematological traits, in addition to reducing the adverse effects of heat stress on chicks treated with thermal manipulation during egg incubation.

Bee pollen administered to broilers’ basal diet at a rate of 20 g/kg diet resulted in a 15.5% increase in average daily gain compared to the control group [78]. This increase may be due to the antibacterial properties of phytogenic compounds and the presence of micronutrients with beneficial effects on metabolism and bird health [79]. This positive effect could also be attributed to bee pollen’s nutritional value as a good supply of protein (approximately 23%), essential amino acids (such as lysine and leucine), fat (approximately 4%), unsaturated fatty acids (such as oleic, linoleic, and linolenic), minerals (such as iron and zinc), and total carbohydrates (approximately 60%). Furthermore, bee pollen stimulates the absorptive and digestive functions of broilers by increasing the absorptive capacity of the intestine by making the villi thicker and longer [74] in relation to a significant increase in body weight gain due to higher protein anabolism and the numerous enzymes supporting the digestive process [80]. Bee pollen (5 g/kg diet) has also been shown in studies to improve growth performance and weight gain in Japanese quails [38]. Bee pollen could be employed in chicken diets as a potential feed additive with prebiotic activity [69]. Farag and El-Rayes [81] discovered reduced feed intake in broiler chickens fed dietary bee pollen diets. This reduction could be attributed to bee pollen products’ high carbohydrate and sugar content, as well as several bioactive compounds that improve feed digestibility, microbial biogenesis, and nutrient utilization efficiency [3]. The beneficial effects of bee pollen on chicken health are supported by the findings of several experimental studies that demonstrated early growth of the bursa of Fabricius and thymus and reduction in the cloacal bursa degeneration as well as the promotion of the splenic immune response in broiler chicks [38,74,78].

According to [41], the inclusion of bee pollen as an oral supplement (250 or 350 mg/kg body weight) can improve growing rabbits’ blood biochemical parameters, feed conversion ratio, and immunity. This supplementation can also increase T_3_ and IGF-1 levels in the plasma without any negative effect on carcass quality. Moreover, bee pollen and propolis combination positively increased rabbits’ body weight, besides decreasing blood cholesterol level, and aspartate aminotransferase/alanine aminotransferase ratio [64]. Zeedan et al. [82] noted that supplementing rabbit diets with 700 mg bee pollen/kg body weight increased body weight and body weight gain as well as improving the feed conversion rate of treated rabbits while decreasing daily feed intake. This decrease in feed intake could be due to the high content of carbohydrates and sugars in bee pollen products, in addition to some bioactive substances that increase nutrient utilization efficiency, feed digestibility, and microbial biosynthesis [4]; thus, it does not make rabbits feel nutritionally deficient. In addition, blood biochemical and immunological parameters, as well as kidney functions were improved in treated groups compared to the control. Furthermore, Attia et al. [64] revealed that bee pollen (200 mg/kg body weight), with or without propolis, can increase the productive and reproductive performance, as well as the economic efficiency of rabbits.

### 2.3. Royal Jelly

Royal jelly is a prominent bee product that is extensively utilized as a natural nourishment for humans and animals due to its high content of essential nutrients. It is high in vitamins B and C, folic acid, and phenolic acids. It is also a good source of minerals. It has various important biological functions in living beings, such as serving as an antioxidant agent, growth promoter, and immunostimulant [9]. Royal jelly’s antioxidant action is mostly due to the presence of polyphenolic substances. It can be utilized to promote growth rate, gastrointestinal health, and immunological response in animals [9,83]. According to Saeed et al. [9], supplementing poultry diets with royal jelly provides an opportunity to maximize profits from safe and high-quality poultry products. Previous studies that have focused on supplementation of poultry diets with royal jelly (50–200 mg/kg) have shown a substantial increase in body weight, egg production, semen quality, and immune response, as well as producing healthier products [9,83,84,85]. Under heat stress, administering royal jelly to growing rabbits can improve body weight gain and feed conversion ratio as well as alleviate the physiological stress caused by heat stress, according to [86]. In agreement, El-Hanoun et al. [13] found that providing heat-stressed bucks with royal jelly (150 mg/kg of body weight) can enhance their physiological functions, particularly liver and kidney activities, with reduced levels of oxidative stress indicators, as well as avoid summer infertility. This treatment improves fertility, blood testosterone levels, libido, sperm production, and sperm quality, while reducing abnormal and dead sperm concentrations. Furthermore, El-Sherbiny [63] showed that the administration of royal jelly (400 mg/0.25 mL water) to rabbit bucks orally during summer months improved their reproductive performance and semen quality as determined by higher testosterone concentration, conception rate, libido, ejaculate volume, and sperm concentration.

### 2.4. Bee Venom

Honeybee venom (also known as apitoxin) is a complex biogenic mixture that is produced in the venom gland of honeybees and has several pharmaceutical and medical properties [87,88,89]. It is composed of water (88%) and various substances, including peptides and enzymes (such as esterase and protease), while melittin (one of the antimicrobial peptides) is the most effective and powerful agent [90,91,92]. It also contains important substances such as apamin and adolapin (polypeptides), which have anti-inflammatory and antibacterial properties [92,93]. Research studies indicate that bee venom and its primary extract (melittin) have the potential to be used as a natural drug to prevent diabetes complications by restraining glycation-induced alteration in the secondary structure and hemoglobin function [94]. They also have high anti-cancer potential by triggering apoptosis and diminishing the cell cycle without significantly damaging normal cells [95]. Animal trials are becoming more common, indicating the safety of venom dosages that are successfully used in these research experiments. Despite that, the usage of bee venom has not been widely known in commercial poultry and rabbit farms.

Bee venom supplementation in drinking water (1 mg/L) had a substantial effect on broiler performance, including body weight, feed intake, and antioxidative capacity, especially during the early stages of life [96]. In the same manner, El-Banna et al. [44] revealed that the use of bee venom in drinking water of broilers up to 2 mg/L can improve productive performance such as body weight, dressing percentage, and carcass traits as well as some internal organs (thymus, liver, and spleen). Bee venom can also stimulate and enhance chicks’ immune responses by being injected with 0.5 mg/bird [97]. This makes bee venom treatment appealing as an alternative to antimicrobial growth stimulants and a good practical approach for use as an alternative to administering antibiotics regularly without any negative effects.

On the other hand, bee venom can be a safe naturalistic growth promoter, antioxidant, immune stimulant, and anti-inflammatory replacement for synthetic chemical drugs in rabbit farms, as injecting 0.3 mg bee venom/rabbit twice weekly improved rabbit productive performance and welfare considerably [4,98]. According to Adel et al. [49], injecting growing rabbits with 0.3 mg/rabbit of bee venom twice a week from 5 to 10 weeks of age resulted in significantly higher feed conversion ratio and body weight compared to the control group, as well as better carcass quality and relative economic efficiency. In agreement, Elkomy et al. [45] mentioned that bee venom, at 2, 4, and 8 mg/kg body weight/day in drinking water, led to a significant increase in body weight and a decrease in feed intake, resulting in improved feed conversion rate and carcass quality of rabbits. They also reported that bee venom positively affects the immunological and antioxidative activities of growing rabbits along with reducing the pathogenic bacteria count in the hindgut.

Bee venom can also be an effective and safe alternative for use in rabbit farms instead of artificial synthetic reproductive enhancers that can impair consumer health [46,47]. It can boost buck semen quality (including sperm concentration (35%), live sperm (6%), and fertility percentage (14%)) and enhance doe reproductive efficiency (including in conception (17%) and fertility rates (10%)) when administered (injection) in small doses (0.1–0.3 mg/kg). It can also improve rabbits’ blood biochemical parameters (such as liver enzyme activities) and antioxidant activity (such as glutathione S-transferase and glutathione peroxidase), as well as their immunological indices (such as IgA, IgG, and IgM) [46,47]. According to El-Speiy et al. [48], using bee venom up to 2 mg/kg body weight/day can improve immunological and antioxidative activities while decreasing harmful microorganisms in the hindgut of weaning rabbits. Bee venom has a positive influence on some biochemical parameters such as total protein, albumin, and globulin concentration in animal blood, as well as stimulating liver enzyme activity (aspartate and alanine aminotransferase) [99]. Furthermore, melittin (the active extract of bee venom) regulates cell membrane modifications by influencing the interactions of lipoproteins that affect the development of these membranes [100]. Additionally, apamin and phospholipase A_2_ that have been found in bee venom have a strong immunoregulatory function, thus bee venom could be effective for several immune diseases [27,101].

## 3. Conclusions

Based on the current results, using bee products, such as propolis, bee pollen, royal jelly, and bee venom, either in the diet, drinking water, or by injection, has many benefits to poultry and rabbits. It is a practical approach that can be applied to poultry and rabbit farms, especially in thermally temperate and subtropical regions. Due to their nutritional and pharmaceutical-beneficial properties, bee products have been proven to improve several physiological, reproductive, antioxidative and immunological responses, along with increasing animal productivity. This makes bee product treatment appealing as a natural promising growth promoter, reproductive stimulant, and immunity enhancer in poultry and rabbits, besides enriching the produced meat with vital bioactive substances. However, more studies and investigations are needed to ensure the safe doses and limits of using these products.

## Figures and Tables

**Table 1:** The bioactive components of bee products.

Bee Products	Bioactive Components	References
Propolis	Resin (50–70%), oil and wax (30–50%), pollen (5–10%), and amino acids (2.9%) such as methionine, phenylalanine, isoleucine, lysine, tyrosine, and tryptophan. Sugar (31%), carboxylic acid (17%), terpenoid (14%), aldehyde (~6%), and hydrocarbon (~6%). Phenolics (13.2 mg/mL), flavonoids (34.5 mg/mL), minerals such as magnesium and calcium, vitamins such as B, C, and E, aromatic and essential oils (10%).	[16,17,18]
Pollen	Protein (23% on average) includes essential amino acids (10%) such as methionine, histidine, threonine, leucine, isoleucine, lysine, valine, phenylalanine, and tryptophan. Digestible carbohydrates (30% on average), sugars (26%) mainly glucose and fructose, and essential fatty acids (~5%).	[19,20,21]
Royal Jelly	Proteins (18%), carbohydrates (15%), sugar (~13%) mainly glucose and fructose, lipids (6%), trace minerals such as copper and iron, water-soluble vitamins such as B and C. Free amino acids such as methionine, phenylalanine, threonine, leucine, isoleucine, lysine, and valine.	[22,23]
Venom	Melittin (50%), phospholipase A_2_ (16%), apamin (2%), adolapin (1%), and hyaluronidase. Amino acids such as histidine, alanine, cysteine, glutamic acid, and tyrosine. Sugars, phospholipids, biogenic amines such as histamine and dopamine, minerals such as calcium, phosphorus, and magnesium, volatile compounds, and pheromones.	[24,25,26,27]

**Table 2:** Summarizes the main results of applying bee products at varied methods and doses on poultry and rabbits.

Bee Products	Administration and Dose	Main Results	References
Propolis	Supplemented at the level of 300 mg/kg of diet	Improves growth performance, antioxidative capacity, and immune response of chickens.	[35]
	Added at the level of 250–500 mg/kg of diet	Lowers the colonization of *Salmonella* spp. and *Escherichia coli* in the cecum and enhances the immunological response of growing rabbits.	[36]
	Administered at the level of 50 μL/kg of body weight	Serves as a natural growth promoter and prevents coccidiosis for growing rabbits.	[37]
Pollen	Administered at the level of 5 g/kg of diet	Improves quail males’ growth performance and body weight gain.	[38]
		Boosts quail hens’ egg production and immunological response.	[39]
	Added at the level of 30 g/kg of quail male diets	Increases body weight and testosterone levels as well as improves reproductive efficiency of quails.	[40]
	Supplemented at the level of 2 g/kg of diet	Decreases feed intake and plasma LDL-cholesterol level as well as improves feed conversion ratio of chickens.	[29]
	Included as an oral supplement at the level of 250 or 350 mg/kg of body weight	Improves growing rabbits’blood biochemical parameters (total protein, T_3_, and IGF-1), feed conversion ratio, and immunity.	[41]
Royal Jelly	Administered at the level of 100–200 mg/kg of diet	Increases laying hen–day egg production rate.	[42]
	Administered at the level of 150 mg/kg of body weight	Improves rabbits’ physiological status including liver and kidney functions and prevents summer infertility.	[43]
	Administered at the level of 200 mg/kg of diet	Maximizes profits from safe and high-quality products of chickens.	[9]
Venom	Added at the level of 2 mg/L of water	Decreases feed intake and plasma LDL-cholesterol level as well as improves feed conversion ratio of chickens.	[29]
	Administered in drinking water up to 2 mg/L	Boosts body weight, dressing percentage, carcass quality, and immunity of broilers.	[44]
	Added at 2, 4, and 8 mg/kg body weight/day in drinking water	Improves feed conversion rate by reducing feed intake and increasing body weight of rabbits.	[45]
	Injecting 0.3 mg/rabbit twice weekly	Improves bucks’ semen quality, blood biochemical parameters, antioxidant capacity, and immune response.	[46]
	Injecting 0.3 mg/rabbit twice weekly	Enhances rabbit does’ reproductive behavior and efficiency as well as liver and kidney functions.	[47]
	Injecting up to 2 mg/kg of body weight/day	Improves antioxidant status, immunity, and bacterial count in the hindgut of weaning rabbits.	[48]
	Injected at 0.3 mg/rabbit twice a week from 5 to 10 weeks of age	Improves feed conversion ratio, body weight, and carcass quality of growing rabbits.	[49]
	*In ovo* injection of 10 µg of melittin/egg on day 18 of incubation	Increases the post-hatch chick’s weight and enhances the immunological indices.	[12]

## Data Availability

The data can be available from the corresponding author upon a reasonable request.
